# Measurement of Neutralizing Serum Antibodies of Patients Vaccinated with Human Papillomavirus L1 or L2-Based Immunogens Using Furin-Cleaved HPV Pseudovirions

**DOI:** 10.1371/journal.pone.0101576

**Published:** 2014-07-07

**Authors:** Joshua W. Wang, Subhashini Jagu, Chenguang Wang, Henry C. Kitchener, Sai Daayana, Peter L. Stern, Susana Pang, Patricia M. Day, Warner K. Huh, Richard B. S. Roden

**Affiliations:** 1 Departments of Pathology, The Johns Hopkins University, Baltimore, Maryland, United States of America; 2 Department of Biostatistics, The Johns Hopkins University, Baltimore, Maryland, United States of America; 3 Department of Oncology, The Johns Hopkins University, Baltimore, Maryland, United States of America; 4 Department of Gynecology and Obstetrics, The Johns Hopkins University, Baltimore, Maryland, United States of America; 5 Woman's Cancer Centre, St Mary's Hospital, Institute of Cancer Sciences, University of Manchester, Manchester, United Kingdom; 6 Paterson Building, Institute of Cancer Sciences, University of Manchester, Manchester, United Kingdom; 7 Laboratory of Cellular Oncology, National Cancer Institute, Bethesda, Maryland, United States of America; 8 Division of Gynecologic Oncology, University of Alabama at Birmingham, Birmingham, Alabama, United States of America; International Centre for Genetic Engineering and Biotechnology, Italy

## Abstract

Antibodies specific for neutralizing epitopes in either Human papillomavirus (HPV) capsid protein L1 or L2 can mediate protection from viral challenge and thus their accurate and sensitive measurement at high throughput is likely informative for monitoring response to prophylactic vaccination. Here we compare measurement of L1 and L2-specific neutralizing antibodies in human sera using the standard Pseudovirion-Based Neutralization Assay (L1-PBNA) with the newer Furin-Cleaved Pseudovirion-Based Neutralization Assay (FC-PBNA), a modification of the L1-PBNA intended to improve sensitivity towards L2-specific neutralizing antibodies without compromising assay of L1-specific responses. For detection of L1-specific neutralizing antibodies in human sera, the FC- PBNA and L1-PBNA assays showed similar sensitivity and a high level of correlation using WHO standard sera (n = 2), and sera from patients vaccinated with Gardasil (n = 30) or an experimental human papillomavirus type 16 (HPV16) L1 VLP vaccine (n = 70). The detection of L1-specific cross-neutralizing antibodies in these sera using pseudovirions of types phylogenetically-related to those targeted by the L1 virus-like particle (VLP) vaccines was also consistent between the two assays. However, for sera from patients (n = 17) vaccinated with an L2-based immunogen (TA-CIN), the FC-PBNA was more sensitive than the L1-PBNA in detecting L2-specific neutralizing antibodies. Further, the neutralizing antibody titers measured with the FC-PBNA correlated with those determined with the L2-PBNA, another modification of the L1-PBNA that spacio-temporally separates primary and secondary receptor engagement, as well as the protective titers measured using passive transfer studies in the murine genital-challenge model. In sum, the FC-PBNA provided sensitive measurement for both L1 VLP and L2-specific neutralizing antibody in human sera. Vaccination with TA-CIN elicits weak cross-protective antibody in a subset of patients, suggesting the need for an adjuvant.

## Introduction

The seminal discovery by zur Hausen that certain oncogenic genotypes of Human papillomaviruses (HPV) typified by HPV16 are the etiologic agents of cervical cancer has led to the commercial development of two preventive vaccines, Gardasil and Cervarix [1]. Their development began with the demonstration that major capsid protein L1 self-assembles into virus-like particles (VLP) [2]. L1 VLP vaccination elicits high titers of type-restricted serum neutralizing antibodies which confer protection from experimental viral challenge after passive transfer of naïve animals [3], [4]. In line with preclinical studies, vaccination of patients with HPV16 L1 VLP also induces type-restricted neutralizing antibodies, suggesting the need for multivalent formulation [5]. As a result, both licensed vaccines contain L1 VLPs derived from HPV16 and HPV18, the oncogenic genotypes that respectively cause circa 50% and 20% of all cervical cancer cases. Gardasil also contains L1 VLP of benign genotypes HPV6 and HPV11, which are the most common cause of genital warts. These L1 VLP vaccines were proven safe, highly immunogenic, and protective against infection and anogenital neoplasia associated with the vaccinal genotypes [6], [7], [8], [9], [10]. However, these vaccines confer limited cross-protective potential towards the most phylogenically-related types and none for the ∼12 other oncogenic HPV types that together cause the remaining ∼30% of cervical cancer cases [11], [12], [13]. A nonavalent prophylactic VLP vaccine being developed by Merck is intended to broaden protection against the remaining oncogenic HPV types, but this complex formulation may be costly to produce, limiting access for low resource settings [14].

An alternative approach to broaden protection is vaccination with the papillomavirus minor capsid protein L2 which induces broadly cross-neutralizing antibodies and protects against experimental challenge with diverse HPV genotypes in animal models [15], [16], [17]. Further, L2-based HPV vaccines can be simply and potentially inexpensively manufactured as a single antigen in bacteria. However, L2 is weakly immunogenic in animals compared to L1 VLP [18], [19], [20]. No clinical studies have examined the ability of L2-based vaccination to protect against natural acquisition of HPV infection, although a few have tested its immunogenicity in patients. For example, the safety and immunogenicity of TA-CIN, a fusion protein of HPV16 E6, E7 and L2 produced in bacteria, has been tested in healthy volunteers and women with high grade vulval intraepithelial neoplasia (VIN), alone or in combination with topical imiquimod or a recombinant vaccinia virus expressing E6 and E7 (TA-HPV) [21], [22], [23]. Vaccination with TA-CIN elicited low titers of HPV16 and HPV18 neutralizing antibodies, and the L2-specific antibody responses in VIN patients were significantly lower than for healthy volunteers [24].

Production of native HPV *in vitro* requires specialized culture conditions, and infection does not have a readily discernible phenotype in animals. Hence, HPV pseudovirus production using codon optimized L1 and L2 genes and the encapsidation of a luciferase marker plasmid to facilitate the detection of infection of 293TT cells or upon vaginal challenge of mice have been used to circumvent these limitations[25], [26]. Using these tools, it was shown that active immunization with L2 immunogens or passive transfer of naïve mice with L2 antisera protects against experimental vaginal challenge with HPV pseudovirus. These antisera often have robust L2 ELISA titers but surprisingly, a low or undetectable neutralization titer when assessed with the standard *in vitro* HPV pseudovirus-based neutralization assay (L1-PBNA) [27], [28], [29], [30], [31]. These observations suggest that vaccination with L2 results in predominantly non-neutralizing antibodies [32] and/or that the L1-PBNA is insensitive for the detection of L2-specific neutralizing antibodies.

Day *et al* identified spatio-temporal differences during the early events of HPV infection *in vivo* versus the *in vitro* infection of 293TT cells resulting in an abbreviated opportunity for furin cleavage of L2, exposure of its neutralization epitopes on the virus surface and thus for L2-mediated neutralization to occur in the L1-PBNA [33], [34], [35]. To enhance the sensitivity for L2-specific neutralizing antibodies, improved neutralization assays have been developed recently [36], [37], [38]. Day *et al* utilized an extracellular matrix, exogenous furin and a different target cell line to ensure L2 neutralizing epitope exposure in their L2-PBNA and better emulate infection *in vivo*. An alternate strategy to improve sensitivity for L2 antisera is the use of a furin pre-cleaved pseudovirion-based neutralization assay (FC-PBNA) in which antibodies prevent the infection of a furin-deficient LoVoT cell line by HPV pseudovirions pre-treated with furin to expose neutralizing epitopes of L2 [38].

While the L2-PBNA and FC-PBNA have shown better sensitivity towards L2-specific neutralizing antibodies in animal sera, they have not been validated using human serum.

Here, we examine the utility of the FC-PBNA for high throughput measurement of both L1 VLP and L2-specific neutralizing antibodies in human sera, and examine it's correlation to the L1- and L2-PBNAs, and protection from vaginal challenge with HPV pseudovirion upon passive transfer of naïve mice with titrated sera of patients vaccinated with L2.

## Materials and Methods

### Ethics Statement

Sera of patients vaccinated with Gardasil (n = 30) as part of their routine clinical care were obtained with written informed consent at the University of Alabama, Birmingham and with the prior permission of the University of Alabama, Birmingham Internal Review Board (X081124003). The studies using human sera were done with the prior permission of the Johns Hopkins University Internal Review Board (NA_00043331). Animal studies were carried out in accordance with the recommendations in the Guide for the Care and Use of Laboratory Animals of the National Institutes of Health and with the prior approval of the Animal Care and Use Committee of Johns Hopkins University (MO13M425). Mice were anesthetized with isoflurane inhalation and euthanized by carbon dioxide asphyxiation.

### Generation of HPV Pseudovirus (PsV) and HPV furin-cleaved Pseudovirus (fcPsV)

A firefly luciferase expression plasmid was employed as the reporter for both HPV PsV and fcPsV. HPV PsV was used in both neutralization assays and for mouse challenge studies. The HPV fcPsVs were used for FC-PBNA. Standard PsV were generated in 293TT cells as described in the protocol (http://home.ccr.cancer.gov/Lco/pseudovirusproduction.htm). For HPV fcPsV, the methodology was employed as described in [38]. Following virus generation and purification, respective virus fractions was serially diluted and tested on 293TT or LoVoT cultures to assess reporter gene expression and determine the highest dilution value prior to reporter signal saturation. This dilution value was used for all subsequent infectivity and neutralization assays. If reporter signal was not saturating at 1∶1000 (as seen with HPV18 PsV and fcPsV), a general guide line was to choose a dilution where signal was ∼100-fold above background. In fcPsV the extent of cleavage of L2 confirmed as ≥70% by Western blot analysis.

### Cell culture and cell lines

All cell lines were maintained in complete DMEM supplemented with 10% FBS, 1X penicillin/streptomycin, 1X Non-essential amino acids, 1X Sodium Pyruvate (Gibco, Life Technologies, Grand Island NY). 2 µg/ml of puromycin was added to 293TTF cells to maintain furin selection and 200 µg/ml of HygromycinB was used to maintain LoVoT selection.

### Human Serum

Sera of 70/72 patients vaccinated with HPV16 L1 VLP were obtained from a completed phase I clinical study [5]. Sera of patients vaccinated with Gardasil (n = 30) as part of their routine clinical care were obtained at the University of Alabama, Birmingham. WHO reference human serum for HPV16 (05/134) and HPV18 (10/140) serology was acquired from NIBSC (Hertfordshire, UK). Sera (n = 19) were obtained pre- and one month post-treatment with the topical imiquimod for 8 weeks and 3 doses of 125 µg of HPV16 E6E7L2 (TA-CIN) at monthly intervals in women with high grade vulval intraepithelial neoplasia (VIN) enrolled in a prior phase II trial [39].

### L1-based Pseudovirus neutralization assay (L1-PBNA), Furin-cleaved based Pseudovirus neutralization assay (FC-PBNA) and the L2-PBNA

Briefly, For the L1-PBNA 293TT cells or for the FC-PBNA LoVoT cells were seeded at 15,000 cells/well in a 96 well plate. 24 hours later, using another 96 well plate, serum test samples were either serially diluted two-fold (L2 sera at starting dilution 1∶50) or three-fold (L1-sera at starting dilution 1∶200 for L1 specific antibody assessment and 1∶50 for L1 cross neutralizing antibodies assessment) in DMEM culture media, and mixed with HPV PsV (at a dilution previously determined for each batch and type by dilution to ∼100x background). In general, approximately 2 to 5-fold more fcPsV was required for the FC-PBNA (e.g 1∶5000 HPV16 PsV or 1∶1000 HPV16 fcPsV). Mixtures were incubated at 37°C for two hours before being added to 293TT or LoVoT cells. The plates were incubated at 37°C for 72 h. Following this, cells were lysed with 30 µL of Cell Culture Lysis Reagent (Promega, Madison WI) for 15 min at room temperature on a rocking platform. The entire lysates were transferred to a 96-well black plate, and luciferase activity was measured by adding 50 µL of luciferin substrate to each well (GloMax-Multi Detection System, Promega, Madison WI). Assays included a neutralizing serum and L2 antibody to assess batch/assay variability and negative control to determine background. For the L2-PBNA, the assay was carried out as per described in http://home.ccr.cancer.gov/lco/L2neut.htm

### ELISA

Maxisorp microtiter 96-well plates (Thermo Scientific Nunc, Waltham MA) were coated with purified HPV16 full length L2 protein with a 6His tag at 500 ng in 100 µL PBS/well. The plates were incubated overnight at 4°C and then blocked with PBS/1% BSA for 1 h at 37°C. Human serum samples were diluted 1∶50 in PBS/1% BSA were then added to the plates in triplicate for 1 h at 37°C. Following this, plates underwent 3 washes with washing buffer (0.01% *v*/*v* Tween 20 in PBS) before HRP-sheep anti-human IgG diluted 1∶5000 in 1% BSA was added to each well and plates were incubated for 1 h at 37°C. After 3 further washes, 100 µL of ABTS solution, 2,2′Azinobis [3-ethylbenzothiazoline-6-sulfonic acid] (Roche, Basel Switzerland) was added to each well for development, and absorbance at 405 nm read using a plate reader Xmark Plus (Bio Rad, Hercules CA).

### Passive transfer of sera and mouse vaginal challenge studies with HPV58

Balb/c mice 6–8 weeks old were purchased from Jackson Laboratories (Maine, USA) and were injected subcutaneously with 3 mg of medroxyprogesterone (Depo-Provera, Pfizer, New York). Three days later, 100 µL of pre-vaccination patient sera or,100 µL, 33 µL or 10 µL of post-vaccinated patient sera was injected intra-peritonally into groups (n = 5) of mice. The following day, each mouse was challenged with 2 µL of HPV58 PsV in 20 µL (2.2×10^9^ Viral Genome Equivalents/mouse). An equal volume of 3% CMC (Carboxymethylcellulose sodium salt, Sigma, St Louis MO, USA) in PBS was added to make a total virus challenge volume of 40 µL per mouse. Mice were anesthetized with isoflurane inhalation to effect prior to administration of virus. Half of the challenge dose (20 µL) was injected into the mouse vaginal vault, followed by insertion of a cytobrush cell collector that was turned both clockwise and counter-clockwise 15 times to induce trauma. After removal of the cytobrush, the remaining half of the inoculum was deposited in the vagina. At 72 h after challenge, the mice were anesthetized again and 20 µL of luciferin (7.8 mg/mL) was deposited to in the vaginal vault. Using a Xenogen IVIS 100 imager, bioluminescence was acquired. Signal intensities were further analyzed using Living Image 2.5 software. A mouse plasma volume of 2 mL based on previous estimates was used to estimate the dilutions for 10 µL, 33 µL and 100 µL injected human sera as 1∶200, 1∶60, and 1∶20 respectively.

### Data analysis and Statistics

For comparisons between all assays, individual human patient sera samples vaccinated with their respective vaccine candidate were analyzed under triplicate L1-PBNA or FC-PBNA assays. To calculate the EC50 value (the reciprocal of the dilution that causes 50% reduction in luciferase activity), the non-linear model Y = Bottom + (Top-Bottom)/(1+10∧((LogEC50-X)*HillSlope)) was fitted to the log_10_ transformed neutralization titers triplicate data using Graphpad Prism 6. The estimated EC50 (which is an average of the 3 triplicate studies is reported as the titer. Patients who had neutralization titers of <1∶50 were assigned an EC50 value  = 1. For assay comparisons, Deming regression[40] and graph plots was applied to the log2 transformed observed EC50 values by using R Version 3.03 with package mcr. The error ratio of the two methods was assumed to be 1. The estimation of the regression model parameters, including intercept and slope, and their bootstrap confidence intervals are reported as well as the Pearson's correlation coefficient (r). For L2-PBNA comparisons, the sparse numbers of positive data resulted in confidence intervals being calculated using the jackknife method. All Deming regression values were rounded off to two decimal places, whereas all EC50 values were rounded to the nearest whole number.

## Results

### Sensitivity of FC-PBNA for WHO reference standards to HPV16/18 antibodies

We first tested the WHO international standards for HPV16 (05/134)[41] and HPV18 (10/140)[42] serology to compare the L1- and FC-PBNA. Our results showed the L1-PBNA and FC-PBNA estimated titers of 120.6 (95% CI = 97.44–149.2) and 146.8 (95% CI = 75.33–285.9) respectively for the HPV16 (05/134) serum. Importantly, these titers were within the range (all titers <1∶200) described for the WHO HPV16 standard when tested previously by eight independent laboratories. We next tested the WHO HPV18 standard serum using both the L1- and FC-PBNAs and obtained titers of 959.4 (95% CI = 840–1095) and 1290 (95% CI = 714–2330) respectively. This EC50 value was also within the range of titers (80–1350) detected by 13 laboratories which previously tested the HPV18 standard. Both standards to 16 and 18 were also tested against different HPV types such as HPV 31 and 45 which resulted in no detectable neutralization at 1∶50 dilution (Data not shown). Together, this indicates that the L1- and FC-PBNA are similarly sensitive in detecting the HPV16 and HPV18 L1 VLP-specific antibody in the WHO standards which were derived by pooling sera from naturally infected individuals.

### Correlation of FC-PBNA and L1-PBNA for detection of neutralizing serum antibodies of patients vaccinated with L1 VLPs

**Figure 1:**
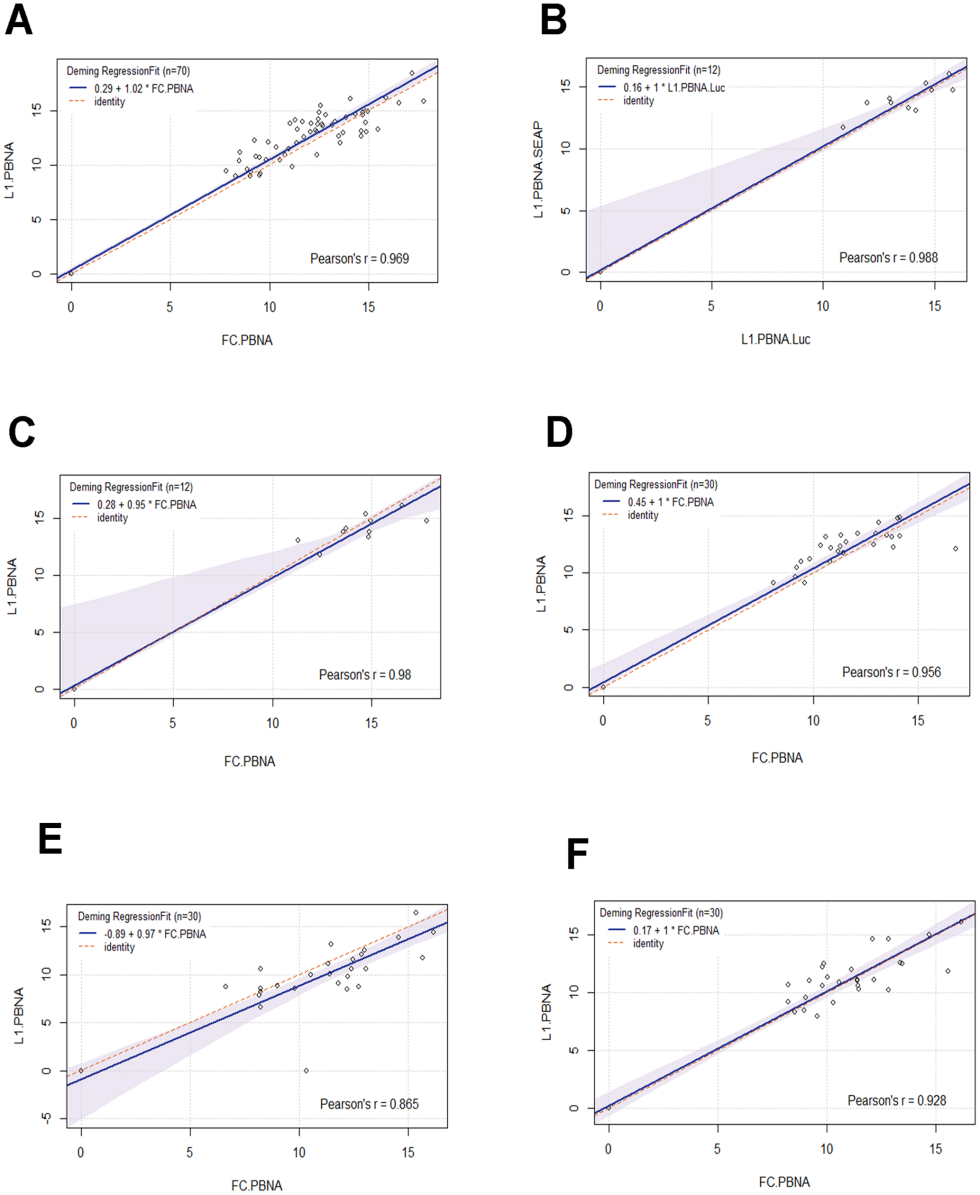
Correlation of neutralization assays. The estimated EC50 values of each patient sample from the respective assays were log2 re-transformed and plotted using R package mcr. Values for Person's r, slope and intercepts were rounded to 2 decimal places. The 0.95-confidence bounds are calculated with the bootstrap (quantile) method. Comparison of HPV16 VLP vaccinated patient sera (n = 70) in FC-PBNA versus L1-PBNA (A). Comparison of L1-PBNA (B) and FC-PBNA (C) with previous findings (n = 12) by Pastrana and colleagues using SEAP-based L1-PBNA [43]. Comparison of n = 30 Gardasil vaccinated patient sera in FC-PBNA and L1-PBNA against HPV16 (D), HPV18 (E) and HPV6(F).

We next compared the L1-PBNA and FC-PBNA using sera from patients vaccinated with HPV16 L1 VLPs or placebo. The first test involved assessing HPV16 neutralizing antibody titers in sera obtained one month following three intramuscular injections of n = 70/72 volunteers enrolled in a Phase I clinical trial of an experimental HPV16 L1 VLP vaccine or placebo (n = 11) [5]. Comparison of methods was performed via Deming regression (See Materials and Methods). Results showed an intercept of 0.29 (95% CI = 0.061,0.67) and a slope of 1.02 (95% CI = 0.97,1.05). In addition, the Pearson's correlation was estimated as r = 0.97 indicating that both methods were very comparable (Figure 1A).

A subset of patients (n = 12) from this study had been previously tested by Pastrana and colleagues when describing the original L1-PBNA which used the secreted alkaline phosphatase reporter system (SEAP L1-PBNA) instead of firefly luciferase [43]. This further enabled us to compare our FC-PBNA results with the titers described in the published study. Firstly, to check if our L1-PBNA which adopts the luciferase reporter system differed in sensitivity from the SEAP system, we compared our luciferase L1-PBNA detected titers with the published titers obtained using the SEAP L1-PBNA. Deming regression of the intercept and slope was 0.16 (95% CI = −0.048, 6.00) and 1.00 (95% CI = 0.586, 1.05). In addition, Pearson's correlation coefficient was calculated r = 0.99 (Figure 1B). Likewise, a Pearson's r = 0.98 was found between the FC-PBNA and the SEAP assay tested with the same sera (Intercept = 0.28 [95% CI = −0.08,7.9], Slope  =  0.95 [95% CI = 0.43,1.01], Figure 1C). Taken together, these results show that the FC-PBNA is comparable to the L1-PBNA and that it is similarly as sensitive in detecting HPV L1 specific neutralizing antibodies.

The performance of the L1-PBNA and FC-PBNA in detecting HPV16, HPV18, HPV6 neutralizing antibodies elicited by vaccination was next investigated using sera of patients (n = 30) who received Gardasil as part of their routine clinical care (Table 1). Once again, the results and subsequent Deming regression analysis showed high correlation between both assays indicating that the FC-PBNA is as sensitive as the L1-PBNA for detecting L1-specific neutralizing antibodies in patient sera. HPV16 (r = 0.96, Intercept  = 0.45 [95% CI = 0.06, 1.90], Slope  = 1.00 [95% CI = 0.85,1.07]) (Figure 1D), HPV18 (r = 0.87, Intercept  = −0.89 [95% CI = −5.09, 0.72], Slope  = 0.97 [95% CI = 0.83,1.31]) (Figure 1E) and HPV 6 (r = 0.93, Intercept  = 0.17 [95% CI = −6.46,1.21], Slope  = 1.00 [95% CI = 0.87,1.10]) (Figure 1F).

**Table 1:** Gardasil patient serum (n = 30) EC50 titers determined with L1-PBNA and FC-PBNA for HPV 6, 16, 18, 31 and 45.

			Mean Neutralization Titers (EC<50)									
Patient	Gardasil Dosage	Length of time between last vaccination and blood draw	HPV 6		HPV 16		HPV 18		HPV 31		HPV 45	
			FC-PBNA	L1-PBNA	FC-PBNA	L1-PBNA	FC-PBNA	L1-PBNA	FC-PBNA	L1-PBNA	FC-PBNA	L1-PBNA
105	0	N/A	<50	<50	<50	<50	<50	<50	<50	<50	<50	<50
119	1	1 month	7352	1210	278	553	300	100	<50	<50	<50	<50
126	1	4 mths	11281	6021	11581	10024	2576	2180	<50	<50	<50	<50
104	1	6 mths	<50	<50	<50	<50	<50	<50	<50	<50	<50	<50
128	1	2 years	<50	<50	<50	<50	<50	<50	109	<50	85	<50
124	2	2 mths	4381	26098	2543	9943	8183	5995	407	572	<50	<50
112	2	1 year	758	249	4457	10826	289	228	<50	<50	<50	<50
130	2	1 year	1054	2635	765	542	897	376	<50	<50	<50	<50
131	2	1 year	26460	33253	<50	<50	<50	<50	<50	<50	<50	<50
121	3	Immediate*	2768	1574	596	1384	5752	2955	<50	<50	<50	303
109	3	2 mths	1514	1934	1536	8955	2701	1055	<50	<50	<50	<50
114	3	4 mths	900	1542	14342	4746	52910	3358	54	79	<50	<50
108	3	1 year	375	322	2522	5102	6849	431	2880	786	<50	<50
111	3	1 year	497	358	1304	5416	3570	536	<50	<50	<50	<50
120	3	1 year	1264	557	3005	6489	4884	900	<50	<50	<50	<50
122	3	1 year	7372	25482	687	1991	1481	986	139	133	<50	<50
129	3	1 year	900	4851	926	2349	300	372	<50	<50	<50	<50
134	3	1 year	2236	4041	2349	3688	2859	9102	102	105	<50	<50
115	3	2 years	2700	2221	13848	8948	8685	1534	<50	<50	<50	1545
116	3	2 years	2856	1259	7528	5627	300	300	<50	<50	<50	<50
118	3	2 years	2700	2099	2740	3294	42235	88975	<50	<50	8084	<50
123	3	2 years	933	5885	17911	28851	7579	4367	<50	192	<50	<50
125	3	2 years	589	2095	566	800	100	416	<50	<50	<50	<50
127	3	2 years	72900	72900	9035	21040	521	466	<50	214	<50	<50
132	3	2 years	527	760	1831	4597	300	1522	<50	<50	<50	1120
133	3	2 years	47870	3719	17849	9236	73676	21269	398	261	7114	2748
113	3	4 years	4601	2208	8022	10883	5408	1517	<50	<50	<50	<50
117	3	4 years	300	603	112791	4431	1303	<50	<50	<50	<50	<50
107	3	5 years	300	1610	1773	2005	4702	364	<50	<50	<50	<50
135**	N/A	N/A	10627	6248	16560	28160	24690	14689	145	149	1657	357

Neutralization titer is based on the reciprocal of the dilution that causes 50% reduction in luciferase activity. Titers were rounded off to the nearest whole number. *Patient 121 was sampled within a week after receiving the third Gardasil dose. **Clinical information on Patient 135 was unavailable.

### No difference between assays in detecting L1 cross-neutralizing antibodies against HPV31 and 45 in Gardasil patient serum

In our initial FC-PBNA characterization using serum that was pooled from ten Cervarix vaccinated mice, we reported that our FC-PBNA was potentially more sensitive in detecting L1-VLP cross-neutralizing titers against non-vaccine HPV types 31 and 45 when compared to the L1-PBNA[38]. To further investigate this finding, a similar comparison was performed using the individual human volunteers vaccinated with Gardasil. The majority of the volunteers failed to mount an *in vitro* neutralization response against these non-vaccine related virus types detectable in either assay (Table 1), although some cross-neutralization was detected against HPV31 and HPV45 (less than 15%). We next looked at the entire data set to assess if patients who responded against either HPV31 or HPV45 typically had higher neutralization titers against HPV16 or HPV18 respectively, but no clear correlation was observed (Table 1). However, because the data set is limited, we cannot exclude this possibility or that at least some of the HPV31 and HPV45 responses were elicited by natural infection with these types.

### Assay by ELISA and passive transfer of L2-specific antibodies in the sera of patients vaccinated with TA-CIN

**Figure 2:**
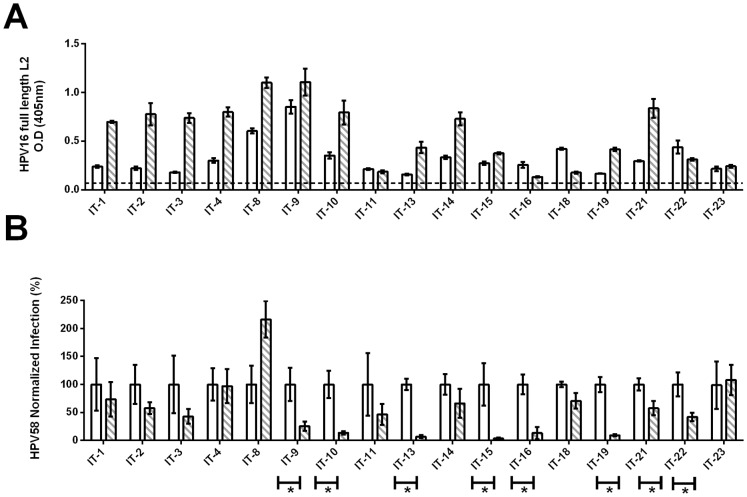
Assessment of TA-CIN sera (n = 17). HPV16 full length L2 ELISA using sera of patients vaccinated with TA-CIN was performed in triplicate and is presented as mean ± Standard deviation (A). Results of *in vivo* passive transfer studies with patient sera. Asterisk indicates significant difference in mean infection of the five mice per group ± standard error against intra vaginal HPV58 challenge observed between pre- and post-vaccinated sera (100 µL per mouse) (B). For both figures, white bars indicate pre-vaccinated serum and hashed bars indicate post-vaccinated serum.

To examine the performance of the neutralization assays for detection of L2-specific antibodies, we utilized pre-immunization and one month post-immunization sera from a phase II study in which 19 vulval intraepithelial neoplasia (VIN) patients who were first treated topically with imiquimod at the lesion site for 8 weeks, and then vaccinated three times at monthly intervals with 125 µg of HPV16 E6E7L2 fusion protein (TA-CIN) with no adjuvant [39]. Since the L1-PBNA may not be fully sensitive towards L2 antibodies, we utilized an ELISA assay with full length HPV16 L2 protein for our initial screen (n = 17/19 patients due to limiting sera) of both pre-vaccinated and post-vaccinated sera to determine if vaccination elicited L2-specific antibodies. A significant increase in the L2 ELISA was observed post-vaccination in 12 of 17 patients tested (Figure 2A).

As the L2 ELISA cannot determine if the antibody response is protective, we next performed passive transfer studies utilizing the *in vivo* mouse challenge model developed by Roberts and colleagues [26]. Although technically demanding and low throughput, it is currently the most sensitive assay for functional antibody assessment [44] and addresses protection from experimental challenge [45]. Importantly, the protective titers determined in these passive transfer studies could subsequently be used for comparison with the *in vitro* neutralizing serum antibody titers detected by the FC-PBNA or L1-PBNA.

As the majority of patients were naturally infected with HPV16 and could therefore have HPV16 L1-specific neutralizing antibodies which can be protective, the passive transfer and PBNA studies were performed with HPV58 pseudovirions. The *in vivo* results (Figure 2B) showed that passive transfer of 100 µL of post-vaccinated serum/mouse from 8 out of 17 TA-CIN patients significantly reduced HPV58 infection after vaginal challenge compared to the pre-immunization serum. It is not clear why vaccination with TA-CIN elicited detectable cross-protective antibody responses in only approximately half of these patients, but this is in line with previous findings that L2 responses in AGIN patients (predominantly VIN patients) vaccinated with TA-CIN were infrequent and of low titer suggesting the requirement for an adjuvant.

### Correlation between PBNA and protection by passive transfer of patient sera for detection of L2-specific antibodies

We next sought to compare the *in vitro* PBNA neutralization titers with these *in vivo* protective titers. Thus the TA-CIN patient sera were tested side-by-side with the L1-PBNA and FC-PBNA for *in vitro* HPV58 neutralization titer assessment (Table 2). The L1-PBNA detected neutralizing titers in only 2 out of 17 patient sera at a 1∶50 dilution. Conversely, the FC-PBNA was able to detect neutralizing antibodies in more of the patient sera (6 out of 17). Importantly, these 6 patients were within the same 8/17 patient sera which were protective in the *in vivo* HPV58 challenge studies by passive transfer of 100 µL, which results in an estimated final dilution of 1∶20 in the mouse (Figure 2B).

**Table 2:** Assessment of sera titers of patients vaccinated with TA-CIN using the L1-PBNA, FC-PBNA and titrated passive transfer assay.

TA-CIN patient no.	Post-Vaccination Sera titer [95% Confidence Interval]		
	L1-PBNA titer	FC-PBNA titer	Passive transfer titer
1	<50 [N/A]	<50 [N/A]	N.D
2	<50 [N/A]	<50 [N/A]	N.D
3	<50 [N/A]	<50 [N/A]	N.D
4	<50 [N/A]	<50 [N/A]	<50 [N/A]
8	<50 [N/A]	<50 [N/A]	<50 [N/A]
9	130 [23 to 736]	113 [13 to 995]	135 [40 to 460]
10	94 [20 to 442]	147 [28 to 775]	101 [50 to 203]
11	<50 [N/A]	<50 [N/A]	N.D
13	<50 [N/A]	1000 [517 to 1934]	923 [350 to 2436]
14	<50 [N/A]	<50 [N/A]	N.D
15	<50 [N/A]	229 [115 to 453]	305 [139 to 671]
16	<50 [N/A]	115 [40 to 326]	191 [88 to 415]
18	<50 [N/A]	<50 [N/A]	N.D
19	<50 [N/A]	512 [161 to 1626]	N.D*
21	<50 [N/A]	<50 [N/A]	N.D*
22	<50 [N/A]	<50 [N/A]	N.D*
23	<50 [N/A]	<50 [N/A]	N.D

Mean titers were rounded off to the nearest whole number and are presented with [95% Confidence Interval]. ND =  Not done. ND* =  Not done due to limited sera available.

**Figure 3:**
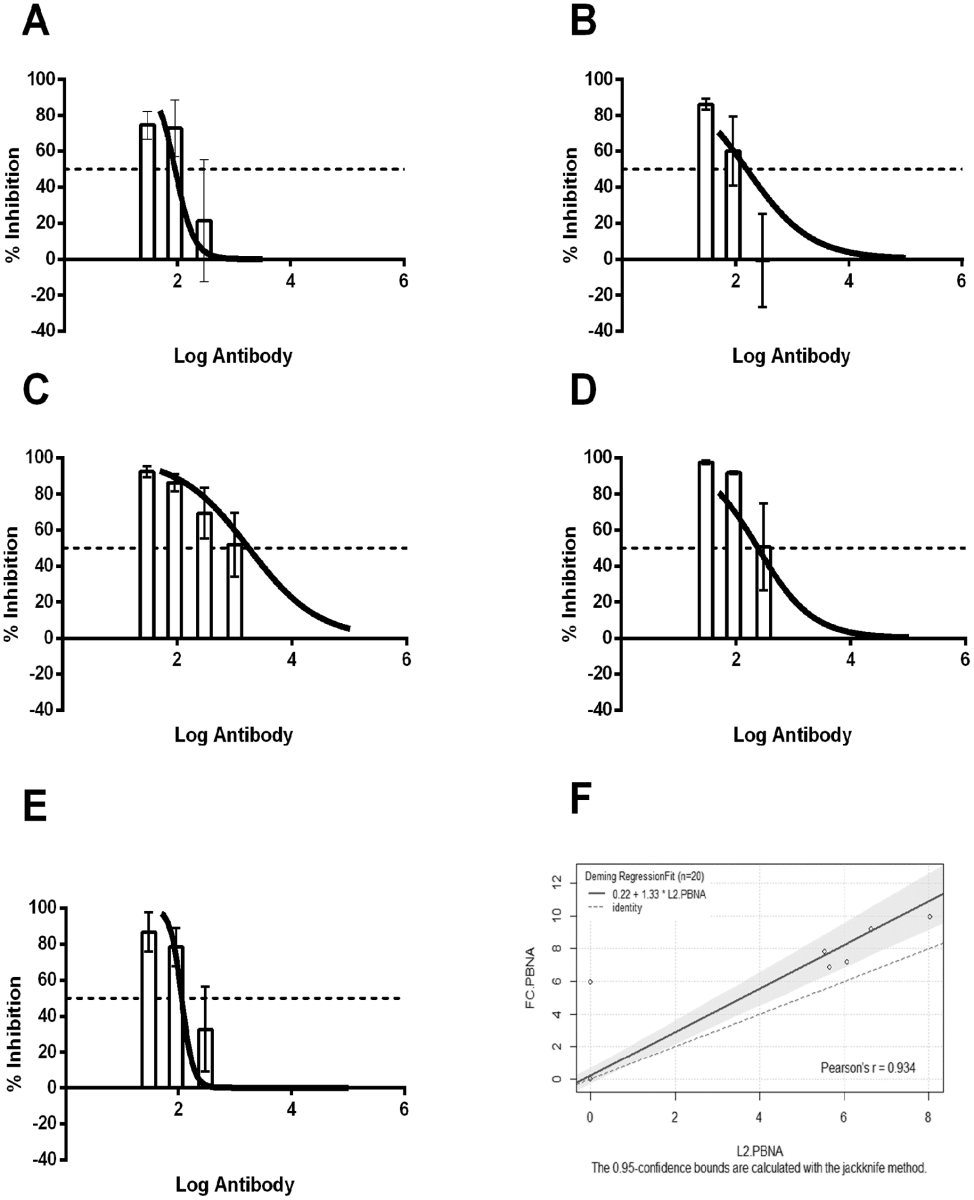
FC-PBNA correlates well with both *in vivo* murine challenge model and L2-PBNA. Comparison of FC-PBNA EC50 fitted *in vitro* neutralization titers curve (black line) with passive transfer studies using titrated dilutions (10 µL, 33 µL, 100 µL) of TA-CIN patient sera (white bars) where IT-9 (A), IT-10 (B), IT-13 (C), IT-15 (D), IT-16(E). For IT-13, sera did not cross 50% inhibition at 10 µL and thus, the experiment with a further 3 µL dilution was repeated to assess *in vivo* inhibition. Comparison of serum titers of patients vaccinated with TA-CIN as detected by FC-PBNA and the L2-PBNA and plotted using R (see text for Pearson's r, slope and intercept) (F).

To further assess analytical sensitivity towards L2-specific neutralizing antibodies, we performed passive transfer studies using titrated amounts of TA-CIN patient sera and correlated the percentage inhibition of HPV58 infection after vaginal challenge to *in vitro* measurements with the FC-PBNA. As the amount of serum for such a study was a limiting factor, only sera from 7 patients could be used. Groups of mice (n = 5) were injected intra-peritoneally with 10 µL, 30 µL, or 100 µL of post-vaccinated serum or 100 µL of pre-vaccinated serum. One day after passive transfer of serum, the mice were challenged with HPV58 pseudovirions. As expected, *in vivo* protection was observed for all five patient sera that were previously positive in both the *in vivo* challenge and FC-PBNA for L2-neutralizing titers. Importantly, the *in vivo* protective titers were very similar in titer value and had overlapping confidence intervals with the *in vitro* neutralizing titers detected by the FC-PBNA (Figure 3, Table 2). In a similar fashion, sera from patients IT-4 and IT-8 which showed an L2-specific response by ELISA but no neutralization titers at 1∶50 dilution were not protective in the titrated passive transfer experiments (data not shown). These findings suggest that the FC-PBNA is able to measure low titers of L2 neutralizing antibodies with greater sensitivity that the L1-PBNA and that its titer measurements were consistent with the protective titers measured using the *in vivo* mouse challenge model.

An *in vitro* PBNA assay with improved sensitivity for L2-specific neutralizing antibodies has been previously reported by Day and colleagues [36]. This L2-PBNA also reported good correlation with passive transfer studies utilizing the *in vivo* mouse challenge model [26]. Using the same sera from patients vaccinated with TA-CIN, we performed the L2-PBNA and compared the titers with those obtained using the FC-PBNA. A Pearson's correlation of r = 0.93 (Intercept  = 0.22 [95% CI = −0.26, 0.70], slope 1.33 [95% CI = 1.15,1.51]) was found between these assays suggesting the methods are comparable in sensitivity and the feasibility of using either assay for detection of L2-specific neutralizing antibodies (Figure 3F).

## Discussion

In several infectious disease models low titers of neutralizing antibody titers are sufficient for protection [45]. Indeed patients are durably protected against HPV18 after vaccination with Gardasil despite titers of antibody to the H18.J4 neutralizing epitope measured by cLIA waning to the background cutoff [46], [47]. Importantly, in the majority of these samples neutralizing antibodies could be detected using the L1-PBNA [48], suggesting the importance of a sensitive and functional assay for immune monitoring of prophylactic vaccination. Similarly, Gardasil has been shown to provide cross-protection against HPV31 despite the inability to detect HPV31 neutralizing antibodies in many patients [12]. This suggests that either low levels of HPV neutralizing antibodies are sufficient for protection or/and that the viral inoculum triggers a rapid recall response that then produces sufficient local levels of antibodies in time to provide complete neutralization. Gardasil does elicit a rapid recall response upon intramuscular injection of a fourth dose, but it is important to note that the challenge dose in this study is systemic, and utilizes adjuvant and a dose likely far greater than natural viral inoculum exposure at the anogenital epithelium [49], and therefore may not be a true reflection of a recall response to natural challenge. Furthermore, this recall response was measured at 1 week post-inoculation [49] and, although the papillomavirus infectious process is slow, post-exposure neutralization is only possible only 8–24 hr later. Thus, a protective recall response would still need to be sufficiently rapid to provide sterilizing immunity. Regardless, since protection can be observed via passive transfer of HPV L1-VLP or L2 antisera in the murine, canine and rabbit challenge models [4], [35], [50], this suggests that such a recall response is not required.

Our study shows for the first time that vaccination with a HPV L2 immunogen in human patients can elicit an immune response that is sufficient to protect naïve animals against vaginal infection. This protection was shown by performing passive transfer studies using sera from individual human patients vaccinated with the fusion protein made from HPV16 L2E6E7 (TA-CIN) (Table 2 and Figure 3). The responses detected to this L2 vaccine were weak suggesting the need for an adjuvant. However a subset of these patient sera that showed a neutralizing response conferred robust protection against a large inoculum of purified virus instilled in the genital tract of the naïve mouse, even at ∼100-fold dilution of the patient sera. Taken together, our observations argue that surprisingly low titers of neutralizing antibodies are sufficient for protection, and is consistent with prior pre-clinical studies using animal antisera or animal neutralizing monoclonal antibodies to L2 or L1 VLP [4], [44], [51].

Since neutralizing antibodies are the relevant immune correlate and low titers are sufficient for protection, the development of sensitive, robust and high throughput assays for HPV neutralizing antibodies regardless of immunogen is important for clinical development of second generation HPV vaccines, especially those based on L2. As mentioned earlier, although the L1-PBNA developed by Pastrana and colleagues is very sensitive for detection of vaccine type L1-specific neutralizing antibodies, the detection of L2-specific neutralizing antibodies and L1-specific cross-neutralizing antibodies has been problematic. As a result of these limitations, several *in vitro* assays including the HT-PBNA[37], the FC-PBNA[38] and the L2-PBNA [36] have been developed to improve the analytic sensitivity for detection of both L1 and L2 neutralizing antibodies over the original L1-PBNA approach. While these assays show clear improvements in sensitivity particularly towards detecting L2-specific neutralizing antibodies in mouse or rabbit sera, further validation with human sera vaccinated with either HPV L1 or L2 immunogens is required. This in turn has been particularly challenging for L2-specific neutralizing antibody detection since natural responses to L2 are rare, and clinical testing of HPV L2-specific vaccines has been limited.

In this study, we attempted to validate our previously developed FC-PBNA's using human patient serum. To do so, we compared its performance to the L1-PBNA. We first compared sensitivity in detecting both L1-VLP specific and cross-neutralizing titers. With respect to the former, both assays were similarly sensitive for detecting HPV L1-specific neutralizing antibodies from natural infection (using the WHO international standards to HPV16 and 18 antibodies) or after VLP vaccination (Figure 1). Together, the results suggest that furin pre-cleavage of PsV does not compromise the key conformational and type-specific L1 neutralizing epitopes [33], [35].

When we previously tested both assays using a single pooled serum from Cervarix-vaccinated mice, the FC-PBNA was more sensitive than the L1-PBNA in detecting HPV31 and HPV45 L1-cross neutralizing antibodies [38] suggesting that furin pre-cleavage might better reveal sub-dominant cross-neutralizing L1 epitopes [52], [53], [54]. To reassess and validate our previous findings, we tested Gardasil patient sera in both assays against HPV31 and 45. However, only a minor fraction of the patient sera had a detectable neutralizing titer for these types (Table 1). This may reflect the lower titers induced in these patients versus mice and also the higher titers elicited by Cervarix versus Gardasil. Additionally, Gardasil may be less protective against HPV45 than Cervarix. This was based previous clinical observations(reviewed in [12]) and our own whereby we observed our FC-PBNA was able to detect cross-neutralizing titers in sera pooled from ten mice vaccinate three times with Cervarix vaccinated mice against HPV31 and 45 but no neutralizing titers (<50) was detected in either assay for these types in a similarly pooled serum from ten mice vaccinated three times with Gardasil (data not shown). Given the demonstrated ability of Gardasil to protect patients and mice from HPV31 [28], [55], this suggests that there is still need to improve the sensitivity of the neutralization assays for detecting L1 cross-neutralizing antibody titers. The application of a more sensitive reporter may be helpful in this regard, as suggested by the use of Gaussia luciferase in the HT-PBNA and its greater sensitivity compared to the L1-PBNA. This is further supported by the report that the HT-PBNA is more sensitive compared to the SEAP-based L1-PBNA and our own findings that the SEAP-based L1-PBNA is highly correlated and similar sensitive as the FC-PBNA using firefly luciferase as the reporter (Figure 1C). Further, the FC-PBNA could potentially be adapted for use with robotics as described for the HT-PBNA to improve sensitivity, reproducibility and throughput.

VIN patients vaccinated with TA-CIN produced weak HPV16 L2-specific serum antibody responses detectable by ELISA (Figure 2). However, ELISA does not discriminate between neutralizing and non-neutralizing L2 antibodies. Indeed, the neutralizing epitopes compromise a very small proportion of the entire L2 sequence, and it appears that non-neutralizing eptiopes toward the the C-terminus of L2 can become immunodominant over neutralizing epitopes at the N-terminus. Hence, although the L2 ELISA is sensitive in detecting L2 antibodies, an *in vitro* neutralization assay is of critical importance to evaluate candidate L2 vaccines. The weak and inconsistent L2-specific responses to 125 µg TA-CIN here, were in line with a previous observation in a different cohort of VIN patients vaccinated with a higher dose of TA-CIN (533 µg) which exhibited weaker responses compared to that of healthy volunteers[39]. Additionally, vaccination of mice with TA-CIN alone also elicits similarly weak L2-specific neutralizing antibodies, but upon use of an adjuvant with TA-CIN, for example the saponin GPI-0100, consistently elicits potent L2-specific neutralizing antibody responses. Notably, these neutralizing antibody titers, although strongly protective, were still lower than those elicited by L1 VLP vaccines [56].

With respect to L2 neutralization, the FC-PBNA was more sensitive compared to the L1-PBNA for detecting the weak L2 neutralizing antibody titers in the sera of patients vaccinated with TA-CIN (Table 2). The FC-PBNA was also more consistent than the L1-PBNA with L2-specific protective titers determined by passive transfer studies in the mouse challenge model. Indeed, the EC50 titers for protection were remarkably similar to the *in vitro* titers for neutralization measured by the FC-PBNA (Table 2 and Figure 3). This observation contrasts a recent study by Longet *et al* wherein the murine model was 200–500-fold more sensitive than *in vitro* assays [44]. However, Longet et al utilized the H16.V5 murine monoclonal antibody or mouse anti-L1 VLP antisera [44], whereas our study tested L2-specific human sera. The discrepancy may reflect preferential stability and/or transport of mouse versus human IgG to the challenge site in the mouse vagina.

We also observed a similar sensitivity for the FC-PBNA and a recently described L2-PBNA assay for L2-specific neutralizing antibodies in human serum (Intercept  = 0.22 [95% CI = −0.257,0.70], Slope  = 1.33 [95% CI = 1.15,1.51]), Pearson's r = 0.93) (Figure 3F). Thus the L2-PBNA and FC-PBNA might be used interchangeably as both are simpler and higher throughput than the passive transfer in the murine challenge model established by Roberts and colleagues [26], although the *in vivo* method is the most sensitive at present and potentially a more biologically relevant approach [44]. However, this requires further validation and highlights the need for additional international standard serum sets for L1 and L2-specific neutralizing antibodies to facilitate comparison of assay formats and validation across different laboratories performing the same assays. We previously reported that the FC-PBNA was >10-fold more sensitive than the L1-PBNA for detection of L2-specific neutralizing antibodies whereas the L2-PBNA reported 100-10,000-fold [36], the limited number of detectable responses and available samples prevents us from determining the absolute magnitude of this difference using human sera. Nevertheless, these findings indicate that the FC-PBNA, as well as the L2-PBNA developed by Day and colleagues [36], are sensitive assays for L1 VLP or L2-specific neutralizing antibody in human serum, and potentially valuable for monitoring immune responses to prophylactic HPV vaccination.
